# In Vitro Biosynthesis of the [Fe]‐Hydrogenase Cofactor Verifies the Proposed Biosynthetic Precursors

**DOI:** 10.1002/anie.202200994

**Published:** 2022-03-30

**Authors:** Sebastian Schaupp, Francisco J. Arriaza‐Gallardo, Hui‐jie Pan, Jörg Kahnt, Georgia Angelidou, Nicole Paczia, Kyle Costa, Xile Hu, Seigo Shima

**Affiliations:** ^1^ Max Planck Institute for Terrestrial Microbiology Karl-von-Frisch-Straße 10 35043 Marburg Germany; ^2^ Laboratory of Inorganic Synthesis and Catalysis Institute of Chemical Sciences and Engineering Ecole Polytechnique Fédérale de Lausanne (EPFL) ISIC-LSCI, BCH 3305 1015 Lausanne Switzerland; ^3^ Department of Plant and Microbial Biology University of Minnesota Twin Cities St. Paul, MN USA

**Keywords:** Acyl Ligands, Biosynthesis, FeGP Cofactor, Guanylylpyridinol, [Fe]-Hydrogenase

## Abstract

In the FeGP cofactor of [Fe]‐hydrogenase, low‐spin Fe^II^ is in complex with two CO ligands and a pyridinol derivative; the latter ligates the iron with a 6‐acylmethyl substituent and the pyridinol nitrogen. A guanylylpyridinol derivative, 6‐carboxymethyl‐3,5‐dimethyl‐4‐guanylyl‐2‐pyridinol (**3**), is produced by the decomposition of the FeGP cofactor under irradiation with UV‐A/blue light and is also postulated to be a precursor of FeGP cofactor biosynthesis. HcgC and HcgB catalyze consecutive biosynthesis steps leading to **3**. Here, we report an in vitro biosynthesis assay of the FeGP cofactor using the cell extract of the Δ*hcgB*Δ*hcgC* strain of *Methanococcus maripaludis*, which does not biosynthesize **3**. We chemically synthesized pyridinol precursors **1** and **2**, and detected the production of the FeGP cofactor from **1**, **2** and **3**. These results indicated that **1**, **2** and **3** are the precursors of the FeGP cofactor, and the carboxy group of **3** is converted to the acyl ligand.

## Graphical abstract

The FeGP cofactor is the prosthetic group of [Fe]‐hydrogenase. The in vitro biosynthesis of this cofactor from synthesized precursors using the cell extract of a mutated strain of *Methanococcus maripaludis* provided evidence for the biosynthesis of the FeGP cofactor from 6‐carboxymethyl‐2‐pyridinols, an organic CO donor, and/or CO and other substances. This method paves the way to analyze the biosynthetic machinery of this H_2_‐activation cofactor.

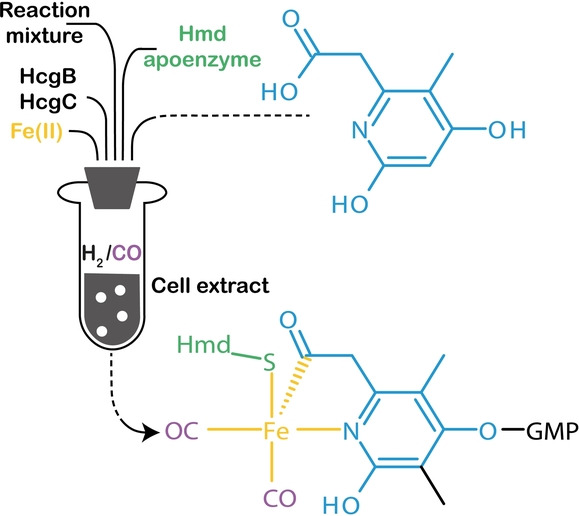

H_2_‐forming methylene‐tetrahydromethanopterin (methylene‐H_4_MPT) dehydrogenase ([Fe]‐hydrogenase or Hmd) catalyzes reversible hydride transfer from H_2_ to methenyl‐H_4_MPT^+^ to form methylene‐H_4_MPT [Eq. 1].^[1]^

(1) $$\mathrm{Methenyl}\text{-}H{}_{4}\mathrm{MPT}{}^{+}+H{}_{2}\vec{\leftarrow }\mathrm{Methylene}\text{-}H{}_{4}\mathrm{MPT}+H{}^{+}\Delta G{}^{\circ }{}^{'}=-5.5\;\mathrm{kJ}\;\mathrm{mol}{}^{-1}$$

This reaction is one of the reduction steps of the methanogenic pathway from H_2_ and CO_2_.^[2]^ [Fe]‐hydrogenase has a prosthetic group, the FeGP cofactor, which contains an iron complex ligated with two CO ligands and a pyridinol derivative; the latter fixes ferrous iron with a 6‐acylmethyl substituent and pyridinol nitrogen (Figure 1).^[3]^ In the enzyme, a cysteine‐thiolate covalently binds to the iron of the FeGP cofactor.^[4]^ The water‐binding site of the iron complex of the FeGP cofactor is proposed to be the H_2_‐binding site for heterolytic cleavage.^[5]^ The FeGP cofactor can be isolated from denatured [Fe]‐hydrogenase in the presence of thiolate compounds: dithiothreitol (DTT), 2‐mercaptoethanol, or acetic acid.[^3a^, ^3e^] The active [Fe]‐hydrogenase holoenzyme is reconstituted by mixing the isolated FeGP cofactor and the heterologously produced apoenzyme.[^3a^, ^6^]

**Figure 1:**
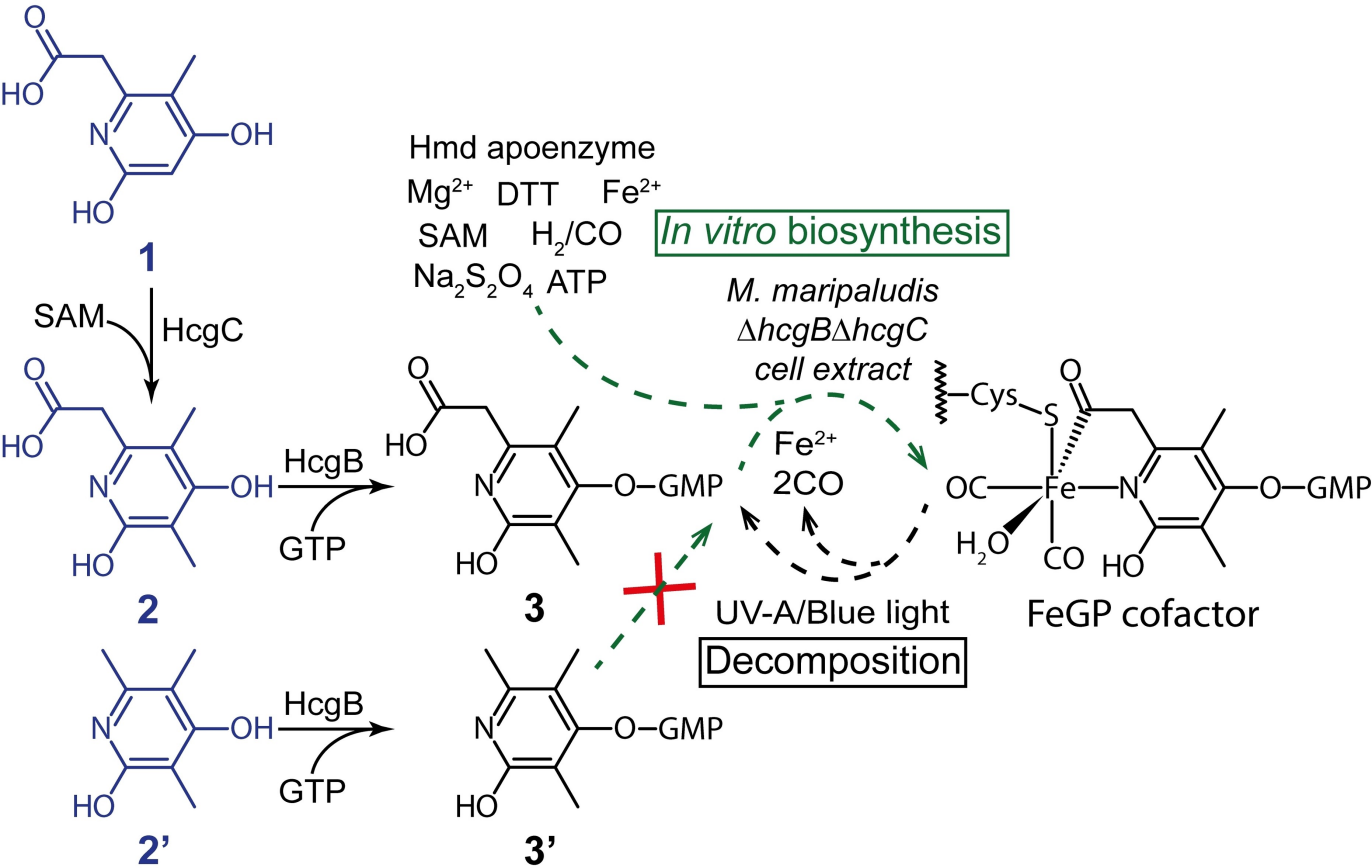
A part of the proposed biosynthetic sequence (from **1** to **3**) and in vitro biosynthesis of the FeGP cofactor. The pyridinol derivatives (**1**, **2** and **2′** in blue) were chemically synthesized. The enzyme reactions, which convert the pyridinol compounds, are shown. **2′** and **3′** did not function as a precursor of in vitro biosynthesis. The acyl group of the FeGP cofactor is hydrolysed to the carboxy group of **3** by light decomposition.

The FeGP cofactor is sensitive to UV‐A/blue light,^[7]^ which causes hydrolysis of the 6‐acylmethyl substituent of pyridinol to form 6‐carboxymethyl‐3,5‐dimethyl‐4‐guanylyl‐2‐pyridinol (**3**) (Figure 1).[^3b^, ^3e^, ^7^] Guanylylpyridinol **3** is a key compound for the study of biosynthesis of the FeGP cofactor (Figure 1).^[8]^ Biosynthesis of the FeGP cofactor is catalyzed by HcgA‐G.^[8b]^ HcgC catalyzes the methylation of 6‐carboxymethyl‐5‐methyl‐2‐pyridinol (**1**) to form **2** using *S*‐adenosylmethionine (SAM) as a methyl donor.^[9]^ HcgB catalyzes the guanylylation of various 4‐hydroxy‐2‐pyridinol compounds, including **2**.[^8b^, ^10^] The observed enzymatic reactions and the cocrystal structure of HcgB and HcgC with pyridinol substrates suggested that 6‐carboxymethyl‐2‐pyridinol derivatives **1**, **2** and **3** are precursors in FeGP cofactor biosynthesis (Figure 1).[^9^, ^10^] Furthermore, the cocrystal structures of HcgE and HcgF with **3** suggested that the carboxy group of the 6‐carboxymethyl substituent is adenylylated by HcgE and that the carboxy group is bound to a cysteine thiolate of HcgF to form a thioester bond.^[11]^ The thioester‐bound carbon of the 6‐substituent of pyridinol of **3** is assumed to be the precursor of the acyl‐iron ligand.^[11]^

However, to determine the structure of the pyridinol precursors, further experiments are required since the acyl group of the FeGP cofactor was biosynthesized from not only CO_2_ but also partially from CO in the gas phase of the culture.^[8a]^ This finding suggests that the acyl ligand is biosynthesized from CO rather than the carboxy group of **3**_._ If CO is the native precursor of the acyl group, then the decarboxylated derivative of **3** (**3′** in Figure 1) could be the real precursor in the biosynthesis of the FeGP cofactor. Here, we report in vitro biosynthesis of the FeGP cofactor using the cell extract of a mutated strain of a methanogenic archaeon, *Methanococcus maripaludis*, which does not produce HcgB and HcgC. This assay provided direct evidence that **1**, **2** and **3** are the biosynthetic precursors of the FeGP cofactor. Furthermore, we showed that the two CO ligands of the FeGP cofactor are biosynthesized from a physiological organic CO donor and from CO in the gas phase during in vitro biosynthesis.

A mutated strain of *M. maripaludis* does not produce HcgB due to the lack of the encoding gene. In addition, this strain does not produce HcgC because the ribosome‐binding site of the *hcgC* gene, which overlaps with the 3′‐region of *hcgB*, was lost together with deletion of the *hcgB* gene. In this report, we refer to this strain as the Δ*hcgB*Δ*hcgC* strain. We confirmed the absence of HcgB and HcgC and the presence of the other Hcg proteins, except for HcgF, in the cell extract of the Δ*hcgB*Δ*hcgC* strain by mass spectrometric proteome analysis (Table S1). Accordingly, the cell extract of the Δ*hcgB*Δ*hcgC* strain did not exhibit [Fe]‐hydrogenase activity. We prepared the standard in vitro biosynthesis solution by adding ATP, SAM, ferrous iron, DTT, sodium dithionite and the [Fe]‐hydrogenase apoenzyme to the cell extract of the Δ*hcgB*Δ*hcgC* strain in 50 mM Tris‐HCl buffer, pH 7.5, containing 5 mM Mg^2+^. In this assay, the biosynthesized FeGP cofactor is bound to the [Fe]‐hydrogenase apoenzyme to form the active holoenzyme. We measured the [Fe]‐hydrogenase activity to determine the amount of the FeGP cofactor biosynthesized in the assay [we measured the reverse reaction of Eq. (1)].

We performed in vitro biosynthesis in the presence of **3** in the standard assay. Compound **3** was prepared by the light decomposition of the FeGP cofactor isolated from [Fe]‐hydrogenase (See Supporting Information). [Fe]‐hydrogenase activity was not observed upon incubation at 40 °C for 1 hour under 100 % N_2_ (Figure 2a). In the case of the same assay mixture under 100 % H_2_, we detected some [Fe]‐hydrogenase activity. The addition of 20 mM formate to the assay also supported in vitro biosynthesis under N_2_. These results indicated that in vitro biosynthesis requires H_2_ or formate as an electron donor, which can be utilized by hydrogenase and formate dehydrogenase.^[12]^ The addition of CO into the gas phase (50 % H_2_ /50 % CO) dramatically increased the [Fe]‐hydrogenase activity. For further studies in this report, we employed 50 % H_2_/50 % CO as the standard gas phase (Figure S1).

**Figure 2:**
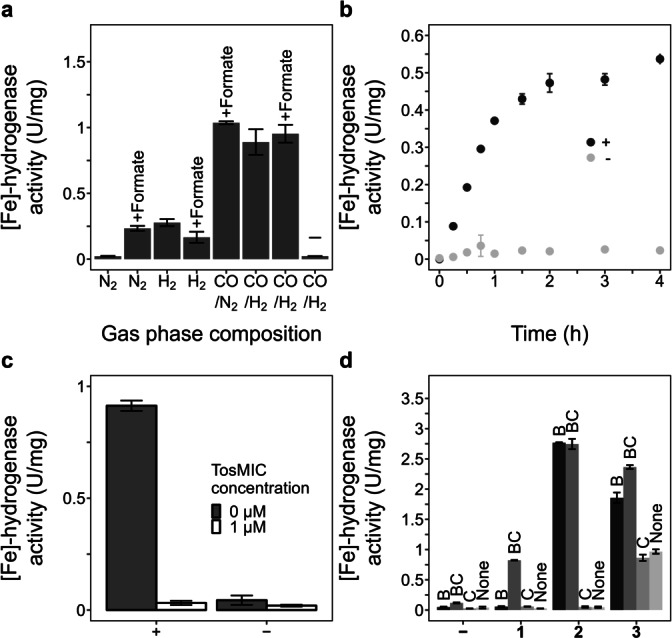
In vitro biosynthesis of the FeGP cofactor. a) Gas requirements. Addition of formate is indicated above the bars. (−) no precursor was added. b) Kinetics of biosynthesis from **3**. Assay with full component (+) and with no precursor (−). c) Inhibition of activity obtained from **3** by TosMIC. d) Activity obtained from the precursors by complementation with HcgB and/or HcgC. Compounds **1**, **2**, **3** or no precursor (−) were added to the assay (abscissa). The Hcg enzymes added are indicated above the bar.

Kinetic analysis indicated that the [Fe]‐hydrogenase activity from **3** increased with incubation time (Figure 2b). The final activity varied among experiments using the cell extracts from different batches of the culture (0.5–5 U mg^−1^). The maximum activity observed was similar to the [Fe]‐hydrogenase activity in the cell extract from the wild‐type strain of *M. maripaludis* under the same culture conditions (4 U mg^−1^). In the absence of **3** or other pyridinol precursors, no [Fe]‐hydrogenase activity was detected. The [Fe]‐hydrogenase activity formed in the in vitro biosynthesis assay was inhibited by 1 μM tosylmethylisocyanide (TosMIC) (Figure 2c), which is a specific inhibitor of [Fe]‐hydrogenase. These results indicated that the FeGP cofactor was synthesized from **3**, and then [Fe]‐hydrogenase holoenzyme was formed in the in vitro biosynthesis assay.

We also used chemically synthesized **1** and **2** as precursors for in vitro biosynthesis. The synthesis of **1** has been described previously.^[9b]^ The synthesis method of **2** is shown in the Supporting Information (Supporting Information, Methods and Figure S2). We observed the formation of [Fe]‐hydrogenase activity from **1** in the presence of GTP, HcgB and HcgC in addition to the standard assay to complement the lacking enzyme reactions (Figure 2d). Removal of HcgB or HcgC from the assay resulted in a loss of activity. Omission of **1** from this assay caused a substantial reduction in the activity; however, we detected residual [Fe]‐hydrogenase activity. These results indicated that some compound **1** accumulated in the cells of the Δ*hcgB*Δ*hcgC* strain. We also observed activity in the in vitro biosynthesis assay from **2**, only when HcgB and GTP were added to the standard assay solution. These experiments confirmed the proposed reaction sequence including **1**, **2** and **3**.

The molecular mass of the FeGP cofactor in in vitro biosynthesis was confirmed by mass spectrometric analysis of the FeGP cofactor extracted from the [Fe]‐hydrogenase holoenzyme biosynthesized in the in vitro assay. We used a Strep‐tagged apoenzyme, and the holoenzyme was isolated using a Strep‐tag affinity column (Supporting Information, Methods and Figure S3).

The FeGP cofactor was extracted from the isolated enzyme and analyzed by a high‐resolution Orbitrap mass spectrometer in negative ionization mode applying a mass resolution of 240 000 coupled to an HPLC using hydrophilic interaction chromatography (Supporting Information). We detected *m*/*z* 635.0234 (the calculated mass is *m*/*z* 635.0207, indicating an error of 4.3 ppm) (Figure S4a). Targeted fragmentation (using collision‐induced dissociation) resulted in fragments of 551.0337 *m*/*z* and 607.0274 *m*/*z* (Figure S4b). This fragmentation pattern was also observed in the samples from the extracted cofactor of [Fe]‐hydrogenase purified from *Methanothermobacter marburgensis*.^[3e]^ In the case of the negative control, which lacks **3** as the precursor in in vitro biosynthesis, we could detect neither the mass of the intact cofactor nor the masses of its fragments. These results indicated that the FeGP cofactor is synthesized during in vitro biosynthesis and that the structure of the FeGP cofactor is the same as that obtained from [Fe]‐hydrogenase of *M. marburgensis*.[^3b^, ^3e^, ^5b^]

To confirm the structure of the biosynthesis precursors, we chemically synthesized the decarboxylated form of **2**, 3,5,6‐trimethyl‐4‐hydroxy‐2‐pyridinol (**2′**) (Figure 1) and used it as a precursor for in vitro biosynthesis. Incubation in the presence of HcgC, HcgB, GTP and **2′** in the standard in vitro biosynthesis solution under 50 % H_2_/50 % CO did not yield [Fe]‐hydrogenase activity. To confirm further, we enzymatically synthesized **3′** from **2′** by the HcgB reaction (Figure S5) and then used it for in vitro biosynthesis. Again, we did not detect any activity (Figure S6). These results disproved the possibility that **3′** is a precursor of FeGP cofactor biosynthesis and supported the idea that the carboxy group of **3** is crucial for biosynthesis.

The requirement for the additives in the in vitro biosynthesis assay was tested by measuring the in vitro biosynthesis from **3** as the precursor. Removal of SAM did not affect in vitro biosynthesis (Figure 3a), and the addition of inhibitors of SAM‐dependent enzymes (1 mM *S*‐adenosylhomocysteine, 5′‐deoxyadenosine and methionine) did not inhibit in vitro biosynthesis. These results suggested that the biosynthesis reaction from **3** under H_2_ and CO does not involve SAM dependent reactions. The omission of ATP substantially decreased the in vitro biosynthesis activity, which is in agreement with the proposed biosynthesis sequence, in which the HcgE‐catalyzed adenylylation of **3** uses ATP.^[11]^ CO titration in N_2_ indicated that half of the maximum in vitro biosynthesis activity was obtained at >0.1 bar partial pressure (Figure 3b). This result indicated that CO can function as a CO donor and/or electron donor. Carbon monoxide dehydrogenase (CODH) can utilize CO as an electron donor.^[14]^ However, the utilization of CO for in vitro biosynthesis appears to be artificial because the physiological CO concentration in methanogen cells should be much lower than the observed concentration. This assumption is also supported by the biosynthesis of the FeGP cofactor in *Methanobrevibacter smithii*,^[8a]^ which lacks CODH.^[15]^ CODH is the only known enzyme that produces CO in methanogenic archaea.

**Figure 3:**
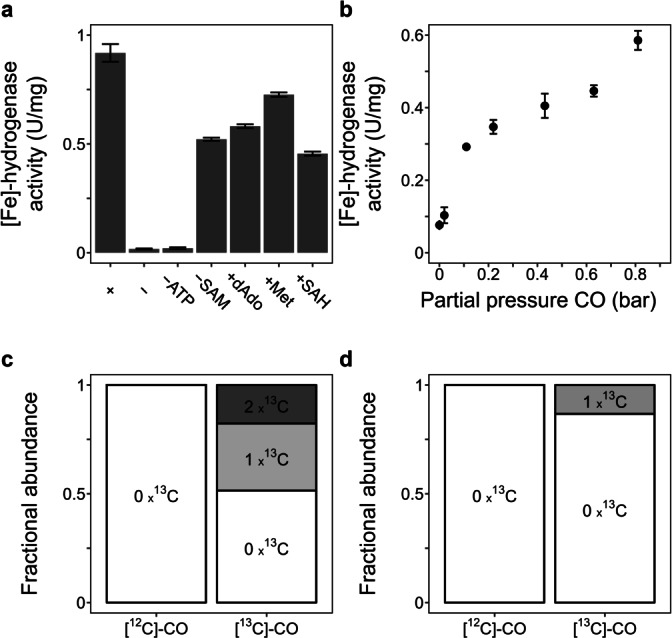
Requirement and incorporation of substances in in vitro biosynthesis. a) Dependency on the substances. (+) Assay with full component of the standard assay. Omission of the substances and addition of potential inhibitors in the absence of SAM, methionine (Met), *S*‐adenosyl‐L‐homocysteine (SAH) and 5′‐deoxyadenosine (dAdo). b) Effect of CO concentrations in N_2_. Partial pressure of CO in the CO/N_2_ mixture in the 1.1 bar gas phase is shown. ^13^C isotope enrichment of the FeGP cofactor (c) and its decomposed product **3** (d) from [^13^C]‐CO as indicated by the fractional abundance of the corresponding ^13^C isotope isomers. The fractional abundance corresponds to the experimentally observed isotope distribution, corrected for the natural isotope abundance contribution using IsoCor (Supporting Information).^[13]^

To test the role of external CO in in vitro biosynthesis, we performed the assay under standard conditions in the presence of **3** under 50 % H_2_/50 % [^13^C]‐CO and analyzed whether [^13^C]‐CO was incorporated into the FeGP cofactor. MS/MS analysis indicated incorporation of [^13^C]‐CO into the CO and/or acyl ligands (Figure S7). After correction for the natural abundance of ^13^C, the isotopic peak profile of the FeGP cofactor generated under [^13^C]‐CO enriched conditions showed that 31 % of the formed molecules carried one ^13^C and that 18 % of the molecules were enriched with two ^13^C (Figure 3c). There was no FeGP cofactor enriched with three ^13^C, which supported that only the two CO ligands originate from external CO. These results were in accordance with the hypothesis that the acyl ligand is biosynthesized from the carboxy carbon of **3**. However, the ^13^C incorporation pattern in in vitro biosynthesis was different from that in previous in vivo labeling experiments using cultivation of a methanogen in the presence of CO, where two or three ^13^C atoms from [^13^C]‐CO were incorporated into the FeGP cofactor.^[8a]^ This discrepancy can be explained by scrambling of the CO and acyl ligands, and metabolic turnover of the cofactor during growth of the cells, where biosynthesis and decomposition of the cofactor occur continuously. To test the scrambling of CO and acyl groups during in vitro biosynthesis, we analyzed the incorporation of ^13^C into decomposed product **3** from the [^13^C]‐CO‐enriched FeGP cofactor. The MS data showed that ≈10 % of **3** was enriched with one ^13^C (Figure 3d). The MS/MS analysis suggested that ^13^C enrichment occurs at the carboxy carbon of **3** (Figure S8). As the carboxy group of **3** is the hydrolyzed product of the acyl ligand,^[3e]^ this result indicates that the acyl ligand is partially enriched with [^13^C]‐CO, which could be the result of scrambling. Scrambling of ^13^C in the acyl/CO ligands of FeGP cofactor mimic compounds has been reported previously.^[16]^

Unexpectedly, 67 % of the CO ligands of the FeGP cofactor are [^12^C]‐CO (see Figure 3c), even though the experiment was performed under 100 % [^13^C]‐CO gas phase, which can be explained by the presence of a CO precursor in the cell extract. This observation is in agreement with the above finding that in the absence of external CO, in vitro biosynthesis occurs under H_2_ or in the presence of formate under N_2_. Most likely, the cell extract contains a physiological CO precursor. A biosynthesis enzyme transiently produces CO from the substance and then uses it for the CO ligand formation. The presence of organic CO precursors is known in the case of other hydrogenase cofactor biosynthesis.^[17]^ External CO can approach the active site and integrate as a CO ligand, as observed in the biosynthesis of [NiFe]‐hydrogenase.^[18]^

In vitro biosynthesis of metallocofactors, for example, the iron–molybdenum cofactor of nitrogenase and the dinuclear iron site of [FeFe]‐hydrogenase has greatly contributed to the elucidation of biosynthetic proteins and precursors.[^17b^, ^19^] Here, we established for the first time the in vitro biosynthesis of the FeGP cofactor, by which we confirmed **1**, **2** and **3** as the biosynthesis precursors by using the chemically synthesized precursors. The results support that the carboxy carbon of 6‐carboxymethyl‐pyridinol **3** is the precursor of the acyl group. In addition, we found that in vitro biosynthesis requires CO, formate or H_2_. These compounds can be utilized as an electron donor in the enzyme systems in the cell extract. These findings are consistent with the proposed acyl forming reaction from the carboxy group and Fe^II^, which requires two electrons.^[11]^ In vitro biosynthesis of the FeGP cofactor will pave the way to analyze the biosynthetic machinery of this unique H_2_ activation cofactor. We are now able to analyze the specific function of the Hcg proteins by in vitro biosynthesis using the Δ*hcg* mutated strains. It is particularly of interest in the studies of HcgA and HcgG, whose enzymatic functions have not been experimentally studied. Furthermore, in vitro biosynthesis allows us to analyze the structure and function of other precursors and electron carriers in the cell extract for biosynthesis of the [Fe] complex.

## Conflict of interest

The authors declare no conflict of interest.

## Supporting information

As a service to our authors and readers, this journal provides supporting information supplied by the authors. Such materials are peer reviewed and may be re‐organized for online delivery, but are not copy‐edited or typeset. Technical support issues arising from supporting information (other than missing files) should be addressed to the authors.

Supporting Information

## Data Availability

The data that support the findings of this study are available from the corresponding author upon reasonable request.
