# Supplementary material for: In Vitro Biosynthesis of the [Fe]‐Hydrogenase Cofactor Verifies the Proposed Biosynthetic Precursors
Source: Angew Chem Int Ed Engl. 2022 Mar 30;61(22):e202200994. doi: 10.1002/anie.202200994 (PMC9314073; doi:10.1002/anie.202200994)
Supplement: Supplementary file 1 — Supporting Information [file ANIE-61-0-s001.pdf]

Angewandte Chemie
Eine Zeitschrift der Gesellschaft Deutscher Chemiker

Supporting Information

In Vitro Biosynthesis of the [Fe]-Hydrogenase Cofactor Verifies the Proposed Biosynthetic Precursors

*S. Schaupp, F. J. Arriaza-Gallardo, H.-j. Pan, J. Kahnt, G. Angelidou, N. Paczia, K. Costa, X. Hu, S. Shima**

Table of Contents

Table of Contents....2
Methods 2
Figure S3. Protein sequence of the Strep-tagged Hmd used for purification of the FeGP cofactor from the *in vitro* biosynthesis. 6
Figure S4. Mass spectrometric analysis of the FeGP cofactor formed in *in vitro* biosynthesis. 7
Figure S6. *In vitro* biosynthesis from the decarboxylated pyridinol precursors **2'** and **3'**. 9
Figure S7. MS/MS spectra of the FeGP cofactor MS species with different ^{13}C content. 10
Figure S8. MS/MS spectra of **3** obtained by light decomposition of the FeGP cofactor produced in *in vitro* biosynthesis. 11
Figure S9. $^{1}H/^{13}C$-NMR trace of **P3**....12
Figure S10. $^{1}H/^{13}C$-NMR trace of **2**....13
Figure S11. HSQC (top) and HMBC (bottom) NMR spectra of **2**. 14
Figure S12. HSQC (top) and HMBC (bottom) NMR spectra of **2'**....15
Figure S13. IR spectra of **1** (**a**), **2** (**b**) and **2'** (**c**). 16
Table S1. Detection of Hcg proteins by proteomics in the *M. maripaludis ∆hcgB∆hcgC* strain. 17
References 17
Author Contributions 17

Methods

Heterologous production and purification of HcgB and HcgC proteins

HcgB and HcgC from *Methanococcus maripaludis* were heterologously produced in *Escherichia coli* BL21(DE3) and purified using His-tag affinity chromatography using Ni^{2+}-charged HiTrap chelating column (GE Healthcare) as described before.[1]

Construction of expression system for [Fe]-hydrogenase apoenzymes

The [Fe]-hydrogenase (Hmd) apoenzyme from *Methanocaldococcus jannaschii* was heterologously produced in *Escherichia coli* as described previously.[2] The *hmd* gene from *M. maripaludis* (WP_011170071.1 or Genbank accession number NC_005791.1) was synthesised and cloned into pET28b(+) vector using NcoI and BamHI (GenScript), in which Hmd was N-terminal Strep-tagged (Figure S3). The plasmid was subsequently transformed into *E. coli* BL21(DE3).

Heterologous production and purification of Strep-tagged [Fe]-hydrogenase apoenzyme from *M. maripaludis*

The *E. coli* strain harbouring the expression vector for production of Strep-tagged Hmd grew aerobically at 37 °C in 2 l LB medium supplemented with 50 µg/ml kanamycin with agitation with a magnetic stirrer (PTFE coated bar). When the optical density (OD) at 600 nm became 1, *hmd* gene expression was induced by addition of 1 mM isopropyl β-D-1-thiogalactopyranoside (IPTG, final concentration) and further cultivation for 4–6 h at 37 °C. The cells were harvested by centrifugation using a Beckmann JLA 10.500 rotor at 7,300 rpm at 4 °C for 20 min and stored until further use at −20°C. The frozen cells were suspended in 40 ml of 100 mM Tris-HCl pH 8 containing 150 mM NaCl, 2 mM dithiothreitol (DTT) and 5% (v/v) glycerol (Buffer A) and lysed by sonication using SONOPULS GM200 (Bandelin) with KE76 tip in ice water with a 50% cycle and 160 W (ten times for 1 min, with 1 min pauses). The cell debris and membrane particles were removed by ultracentrifugation using a Sorvall T647.5 rotor at 30,000 rpm at 4 °C for 30 min. The supernatant was applied to a 5 ml StrepTrap column equilibrated with buffer A. The column was washed with 10 column volumes (CV) of buffer A and then the apoenzyme was eluted by buffer A containing 2.5 mM desthiobiotin. The purified protein was desalted with buffer A by HiPrep 26/10 desalting column, concentrated, frozen in liquid N_2 and stored at −75 °C until further use.

Synthesis of 6-carboxylmethyl-5-methyl-4-hydroxy-2-pyridinol (1), 3,5,6-trimethyl-4-hydroxy-2-pyridinol (2') and 6-carboxylmethyl-3,5-dimethyl-4-hydroxy-2-pyridinol (2)

Compound **1** was synthesized following the literature.[1] The IR spectrum is shown in Figure S13a. Compound **2** was synthesized from **2'** (Figure S2). Compound **2**' was synthesized following the literature.[3] HSQC- and HMBC-NMR (Figure S12). Infrared spectrum (Figure S13c).

Step 1: To the solution of compound **2**' (1.0 g, 6.5 mmol) in DMF (fully dissolved) was added NaH (60% w/w, 286 mg, 1.1 eq) at room temperature. The solution was stirred for 2 h at room temperature. Then chloromethyl methyl ether (MOMCl, 624 mg, 1.2 eq) was added. The mixture was stirred at room temperature for another 8 h. Again NaH (60% w/w, 312 mg, 1.2 eq) was added at room temperature. After stirring for 2 h at room temperature, MOMCl (728 mg, 1.4 eq) was added and the reaction was stirred at room temperature overnight. Na_2CO_3 solution was added to quench the reaction and the mixture was extracted with DCM. After purification by silica column chromatography using hexane/EtOAc as the eluent, compound **P2** was obtained as oil (1.4 g, 89%).
^{1}H NMR (400 MHz, Chloroform-*d*) δ 5.53 (s, 2H), 4.98 (s, 2H), 3.58 (s, 3H), 3.40 (s, 3H), 2.37 (s, 3H), 2.07 (s, 3H), 2.04 (s, 3H) (Figure S9).

Step 2: Compound **P2** (482 mg, 2.0 mmol) was dissolved in dry THF (5 mL) and cooled to −78 °C. Lithium diisopropylamide (LDA) (2 M in THF, 2.9 mL, 2.9 eq) was added slowly and the mixture was stirred at this temperature for 2 h. Then, dimethyl carbonate (216 mg, 1.2 eq) in THF (2 mL) was added dropwise. The reaction was stirred for another 30 min and quenched with water. The mixture was extracted with Et_2O and purification by silica column chromatography using hexane/EtOAc as the eluent, product **P3** was obtained as oil (100 mg, 17%). ^{1}H NMR (400 MHz, Chloroform-*d*) δ 5.51 (s, 1H), 4.98 (s, 1H), 3.73 (s, 1H), 3.70 (s, 1H), 3.59 (s, 1H), 3.50 (s, 1H), 2.13 (s, 1H), 2.13 (s, 1H). ^{13}C NMR (101 MHz, $CDCl_3$) δ 171.12, 163.51, 159.39, 147.81, 119.50, 112.02, 99.17, 91.81, 57.66, 57.08, 51.92, 41.54, 11.87, 9.49.

Step 3: NaOH (615 mg, 5 eq) in H_2O (20 mL) was added to the solution of compound **P3** (920 mg, 3.1 mmol) in MeOH/THF (40 mL / 20 mL). The mixture was stirred at room temperature for 5 h until TLC showed no starting material remained. Aqueous HCl solution (~3 M) was added to adjust the pH to 1. All the volatile was then removed by vacuo. MeOH/EtOAc (30 mL / 30 mL) was added and filtered to remove some dissolved salts. After concentration and dryness, DCM (20 mL) was added. Then CF_3COOH (20 mL) was added at 0 °C and the mixture was stirred at this temperature for 2 h. After stirring at room temperature for another 5 h, the mixture was concentrated. The residue was stirred with MeOH (2 mL) and Et_2O (30 mL) for 30 min at room temperature. **2** was obtained as white solid after filtration (520 mg, 86%). ^{1}H NMR (400 MHz, DMSO-d_6) δ 11.57 (s, 2H), 9.32 (s, 1H), 3.50 (s, 2H), 1.84 (s, 3H), 1.82 (s, 3H) (Figure S10). ^{13}C NMR (101 MHz, DMSO) δ 170.42, 163.14, 162.16, 135.31, 106.18, 105.02, 35.78, 10.32, 9.04 (Figure S10). HSQC- and HMBC-NMR (Figure S11). Infrared spectrum (Figure S13b). HRMS(ESI/QTOF) *m/z* [M - H]$^-$ calculated for $C_9H_{10}NO_4^-$ 196.0615; found 196.0619.

Synthesis and purification of 3,5,6-trimethyl-4-guanylyl-2-pyridinol (3'**)**
Compound **3'** was enzymatically synthesized from **2** and GTP by the reaction catalyzed by heterologously produced HcgB from *M. maripaludis*. The reaction solution containing 100 µM **2**, 5 mM GTP/$MgCl_2$ and 10 µM HcgB in 50 mM MOPS/KOH pH 7 was incubated for 48 h at 40 °C. The reaction was stopped by acidification with 40 µM acetic acid and then the protein was removed by centrifugation using Eppendorf MiniSpin plus at 14,500 rpm at room temperature for 20 min followed with filtration (0.22 µm pore size). The sample was concentrated by evaporation at 4 °C and applied to a HPLC system equipped with a Synergie 4 µm Polar-RP 80 Å column (Phenomenex, Aschaffenburg) with a water/methanol gradient. The fraction containing the sample were collected and concentrated by evaporation at 4 °C.

Purification of 6-carboxymethyl-3,5-dimethyl-4-guanylyl-2-pyridinol (3**) from *Methanothermobacter marburgensis***
M. marburgensis was cultivated hydrogenotrophically in a medium with low nickel concentration using 10 l fermenter as described previously.[4] One hundred gram of *M. marburgensis* cells were suspended in 50 mM potassium phosphate buffer pH 7. A crude extract was prepared by sonication using SONOPULS GM200 (Bandelin) with KE76 tip in ice water with a 50% cycle and 160 W (six times for 8 min, with 7 min pauses). A protein fraction-containing [Fe]-hydrogenase was obtained by ammonium sulphate precipitation and dialysis as described elsewhere.[4] Until this step, the preparation was performed anaerobically under 95% N_2/ 5% H_2 atmosphere in an anaerobic chamber (Coy Laboratory Products, Grass Lake, MI). After dialysis, the FeGP cofactor was extracted from the partially purified [Fe]-hydrogenase fraction under air by addition of a mixture of 60% methanol, 1 mM 2-mercaptoethanol and 1% ammonia solution (final concentrations) under room light. Denatured protein was removed by ultrafiltration (10-kDa cut off) and the solution was dried by evaporation at 4 °C. After dissolving in water containing 1% NH_3, the product was loaded on an anion exchange chromatography (5 ml HiTrap Q HP column). After wash of the column with 5 CV water containing 1% NH_3, the adsorbed compounds were eluted with linear gradient of NaCl (0−1 M) in water containing 1% NH_3. Compound **3** was eluted at 0.35 M NaCl concentration and further purified by HPLC using a Synergi 4m Polar RP 80A column (250 mm × 4.6 mm, Phenomenex) with a water/methanol gradient. The purified **3** was finally concentrated by evaporation at 4 °C.

Cultivation of *Methanococcus maripaludis*
Methanococcus maripaludis ΔhcgB strain was cultivated in the 37 °C cultivation room using a modified medium[5] with sodium formate as substrate under 80% N_2 / 20% CO_2 with Tris as additional buffer component[6] in 5 l or 500 ml scale to an OD at 600 nm of 0.7-0.9. The actively growing cells were anaerobically harvested by a continuous-flow centrifuge (Heraeus 3049 continuous flow rotor at 15,000 rpm at 4°C), resuspended in medium again and sedimented by centrifugation (Beckmann JLA 10.500 rotor at 7,300 rpm and 4°C). The use of the culture medium for resuspension aimed to avoid lysis of the cells in low salt concentration buffer solutions. The cell pellets were finally anaerobically resuspended in a lysis buffer: 50 mM Tris/HCl pH 7.5, 5 mM $MgCl_2$ and 2.5 U/ml DNaseI, to a final concentration of 0.5 g cells/ml buffer. The 1 ml aliquots were frozen in liquid N_2 and stored until use at −20°C. The frozen samples were anaerobically thawed on ice. Unbroken cells and membrane particles were removed by ultracentrifugation using a Sorvall TFT-80.4 rotor at 35,500 rpm and 4°C for 1 h. This supernatant is designated as cell extract and used for *in vitro* biosynthesis assay (see below).

Proteome analysis

Cell pellets were lysed and reduced by tris(2-carboxyethyl)phosphine (TCEP) in the presence of deoxycholate (DOC) at 90 °C for 10 min. After that it was incubated at 25°C for 30 min in ammonium bicarbonate pH 8.2 iodoacetic acid (IAA) and then digested overnight at 30 °C with trypsin, MS approved (Serva). Before LC–MS analysis, samples were desalted using C18 microspin columns (Nest Group) according to the manufacturer's instructions. Dried and reconstituted peptides were then analyzed using liquid-chromatography-mass spectrometry carried out on a Orbitrap Exploris 480 instrument connected to an Ultimate 3000 RSLC nano and a nanospray ion source (all Thermo Scientific). Peptide separation was performed on a reverse phase HPLC column (75 µm x 40 cm) packed in-house with C18 resin (2.4 µm; Dr. Maisch) with a 135 min gradient (formic acid / acetonitrile). MS data were searched against an in-house *Methanococcus maripaludis* S2 protein database using SEQUEST embedded into Proteome Discoverer 1.4 software (Thermo Scientific).

***In vitro* biosynthesis of FeGP cofactor**

We prepared the sample solution in a 1.5 ml Eppendorf plastic cup under 95% N_2/ 5% H_2 atmosphere in an anaerobic chamber (Coy). Twelve µl of a mixture of 100 mM $Fe(SO_4)_2(NH_4)_2$, 100 mM DTT, 200 mM sodium dithionite, 500 mM $MgCl_2$ and 630 mM **3** was added to 200 µl cell extract and then, 5 µl of 250 mM *S*-adenosyl methionine, 2 µl 250 mM ATP and lastly 5 µl 500 mM heterologously produced Hmd apoenzyme from *M. jannaschii* were added. Addition of the Hmd apoenzyme in the earlier points made precipitates. The solution was transferred to a vial with a rubber stopper containing 50% H_2 /50% CO or otherwise described atmosphere. The solution was incubated at 40 °C for 1 h or at room temperature for at least 3 h and the activity of [Fe]-hydrogenase was determined. The enzyme activity measurements were performed mostly in duplicates. The data for Figure 2a and 3a are triplicates. The activity was variable between the experiments using the cell extracts from different batches of the culture; therefore, the data sets shown in each panel were obtained using the same cell extract.

[Fe]-hydrogenase activity assay

[Fe]-hydrogenase was determined by photometrically measuring the conversion of methylene-tetrahydromethanopterin (methylene-H_4MPT) to methenyl-H_4MPT^+ in 120 mM potassium phosphate buffer at pH 6 containing 1 mM EDTA in 1 ml cuvettes (1 cm light pass) in a total volume of 0.7 ml assay solution in the presence of 20 µM methylene-H_4MPT under 100% N_2.[4] Methylene-H_4MPT was prepared as described previously by spontaneous reaction of formaldehyde with H_4MPT.[4] Increase rate of the absorbance at 336 nm was recorded and the activity was calculated using the extinction coefficient of methenyl-H_4MPT^+ (ε336 nm = 21.6 $mM^{-1}\cdot cm^{-1}$). One U was defined as the activity producing one µmol of methenyl-H_4MPT from methylene-H_4MPT per min. Activities are given as U/mg protein in the assay. The protein concentration was assayed using Bradford method using the dye solution ROTI®Quant (Carl Roth) using bovine serum albumin (Bio-Rad Laboratories) as standard. The range of the protein concentrations in the assay was between 15–25 mg/ml.

Preparation of the FeGP cofactor from *in vitro* biosynthesis solution for mass spectrometric analysis

To obtain the purified FeGP cofactor for high resolution Orbitrap mass spectrometer analysis, the standard *in vitro* biosynthesis assay was performed in the presence of 40 µM Strep-tagged Hmd apoenzyme from *M. maripaludis* in a volume of 5 ml. After completion of the *in vitro* biosynthesis assay, 1 U avidin (per ml of the *in vitro* solution) was added and then the solution was diluted twofold with 150 mM Tris-HCl pH 8 containing 100 mM NaCl. The reconstituted holo-Hmd was purified from this solution with the same protocol as for the purification of Strep-tagged Hmd expressed in *E. coli*, except for that buffer B did not contain 5% glycerol. Just after the Streptag column purification, the FeGP cofactor was extracted from the purified enzyme by addition of 60% methanol, 1 mM 2-mercaptoethanol and 1% NH_3 (final concentration) and incubated at 40 °C for 15 min, and then the extracted solution was filtrated (10 kDa cut off). The filtrate was concentrated by evaporation at 4 °C and the dried sample was dissolved in 400 µl of 10 mM $(NH_4)_2(CO_3)$ pH 9 containing 1 mM 2-mercaptoethanol. The sample was filtered by a 0.22 µm filter before analysis by mass spectrometry.

Mass spectrometric analysis

Mass spectrometric determination of the FeGP cofactor was performed using a HRES-LC-MS. The chromatographic separation was performed on an Thermo Scientific Vanquish HPLC System using a polymer based ZICpHilic (Sequant, 150 × 2.1 mm, 5 µm, Merck) equipped with a 20 × 2.1 mm guard column of similar specificity at a constant eluent flow rate of 0.25 ml/min and a column temperature of 40 °C with eluent A being 10 mM ammoniumhydroxyde in water adjusted to a pH of 9.8 and eluent B being acetonitrile (Honeywell) The injection volume was 2 µl. The elution profile consisted of the following steps and linear gradients: 0 – 3 min constant at 95 % B; 3 – 10 min from 95 to 20 % B; 10 – 20 min constant at 20 % B; 20 – 20.1 min from 20 to 95 % B; 20.1 – 30 min constant at 95 % B. Ionisation was performed using a high temperature electro spray ion source at a static spray voltage of 3300 V, Sheath gas at 35 (Arb), Auxilary Gas at 7 (Arb), and Ion transfer tube and Vaporizer at 300 and 275 °C. Full Scan measurements were conducted applying an orbitrap mass resolution of 240 000 without using quadrupole isolation in a mass range of 100 − 642. Data was saved in full profile mode. Targeted fragmentations measurements were performed at similar chromatography and ionisation settings, but using a quadrupole isolation of the target ion in a window of 0.4 *m/z*. Collision induced dissociation was performed in the ion routing multipole with a relative collision energy of 5 %. Fragments were detected using the orbitrap at a predefined mass resolution of 60 000 in the range between 100 and 640. For the analysis of the amount of incorporated ^{13}C in the

[^{13}C]-CO labelling experiments the peak area of the extracted ion chromatogram of the calculated ion mass ± 5 ppm was integrated for each isotopologue and subsequently the natural isotope abundance of ^{13}C was subtracted using the IsoCor software.[7]

Matrix assisted laser desorption ionization – time of flight mass spectrometry (MALDI-TOF-MS) was performed as described previously using a 4800 Proteomics Analyzer (Applied Biosystems/MDS Sciex).[1]

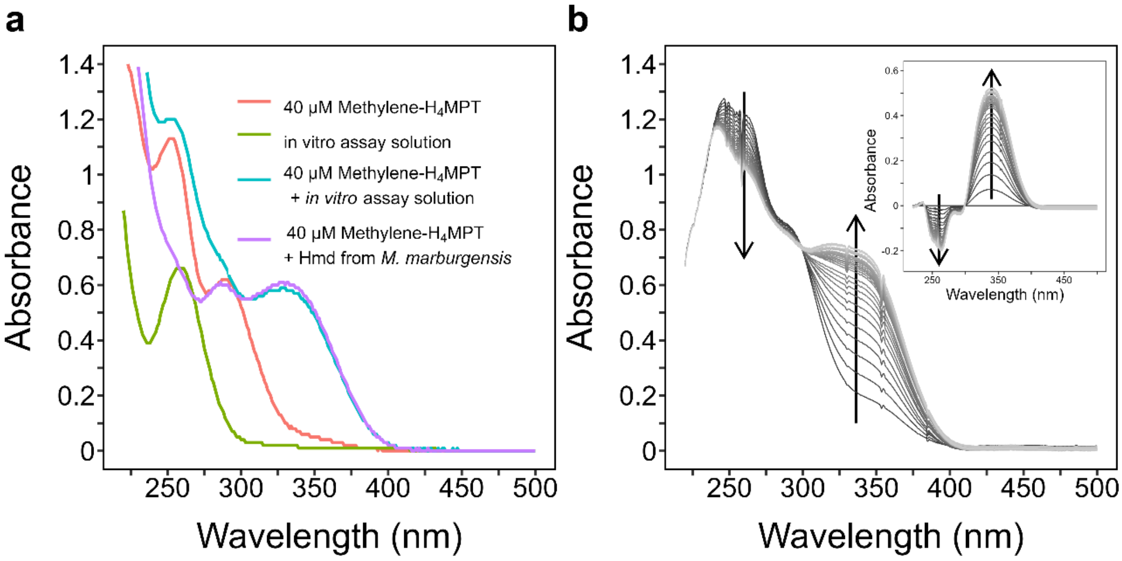

Figure S1. Conversion of the UV-Vis spectrum upon dehydrogenation of methylene-H4MPT catalyzed by [Fe]-hydrogenase (Hmd) produced in the *in vitro* biosynthesis assay. (a) UV-Vis spectra of methylene-H4MPT (red), after the reaction with Hmd formed in the *in vitro* biosynthesis assay (cyan), after the reaction with Hmd purified from *M. marburgensis* as a positive control (violet), and the *in vitro* biosynthesis solution without methylene-H4MPT as a negative control (green). The peak observed at 336 nm indicates the presence of methenyl-H4MPT formed in the solutions. (b) Time-resolved UV-Vis spectrum changes by the reaction of Hmd produced in the *in vitro* biosynthesis assay. The spectra were recorded at each 10 s. The differential spectra were calculated by subtraction of the initial spectrum (Inset). Arrows indicate the direction of the change. The enzyme reaction assays were performed at the standard condition using 1 cm light pass cuvette in the presence of 40 μM methylene-H4MPT. The reaction started by addition of 25 μl 50-fold diluted *in vitro* biosynthesis solution.

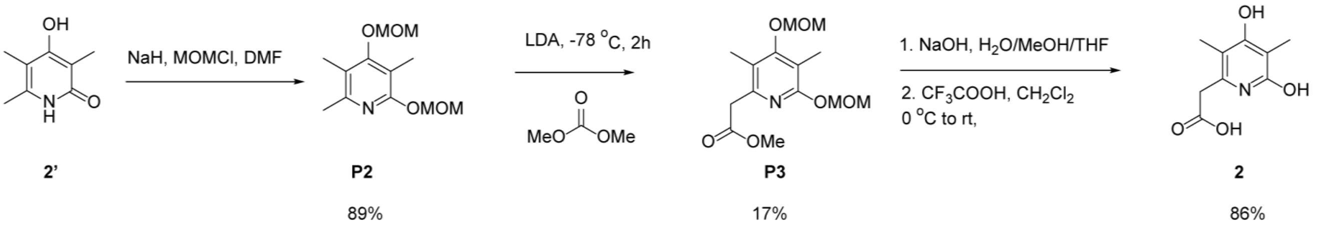

Figure S2. Synthesis of compound **2**.

MASWSHPQFEKSSGLVPRGSKVAILGAGCYRTHAASGITNFSRASQVAKEAGIPEIAMTHSTITMGAELLHLIPEITEVVVSDPCFAEEPGMVVL
DQFDYKAVMEAHLAGDAEKVMPEIREAVKAKAKETPKPPKGCIHFVHPETVGLKVTASDVEAVKDADIVITWLPKGGSQPAIIEKFASEIKKGAIV
THACTIPTPKFAKIFKDLGRDDLNIIAYHPGAVPEMKGQAFLSEGLADAEKVEEFYCMAKTARGEAFKMPANLISPVCDMGSAVTAPVYAAILAY
RDAVTQILGAPADFAQMMADEAISQILDLMRNEGIKNMEDKLNPKALTGTADSMCFGPLADILPASLKVLEKHANENKCECGCSIKP

Figure S3. Protein sequence of the Strep-tagged Hmd used for purification of the FeGP cofactor from the *in vitro* biosynthesis.

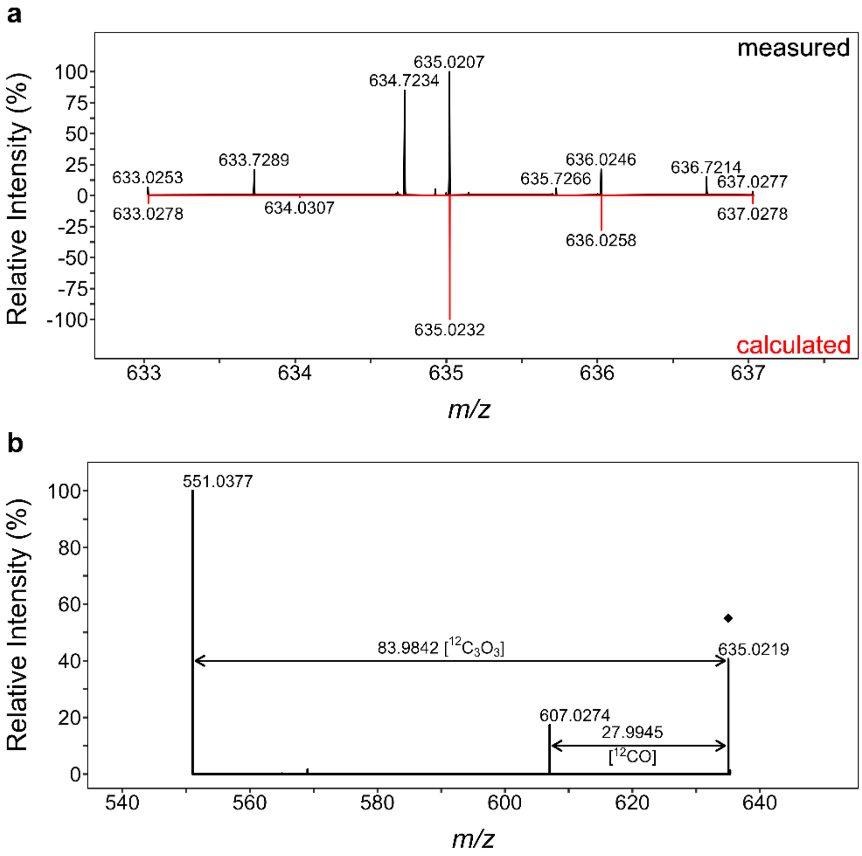

Figure S4. Mass spectrometric analysis of the FeGP cofactor formed in *in vitro* biosynthesis. (a) Comparison of measured and calculated isotope pattern for the FeGP cofactor. (b) MS/MS spectrum of 635 *m/z* species of the FeGP cofactor ($C_{21}H_{21}N_6O_{12}P_1Fe_1$, $[M\text{-}H]^-$ calculated = 635.0232 *m/z*). For all spectra ions in the range of $[M\text{-}H]^- \pm 5$ ppm were selected for collision-induced dissociation. Diamond designates the precursor ions. The 607.0274 *m/z* species corresponds to a species, which lost one CO unit. The 551.0377 *m/z* species corresponds to a species that lost three CO units. This MS/MS pattern suggests that two CO ligand and one acyl ligand dissociated as three CO units.

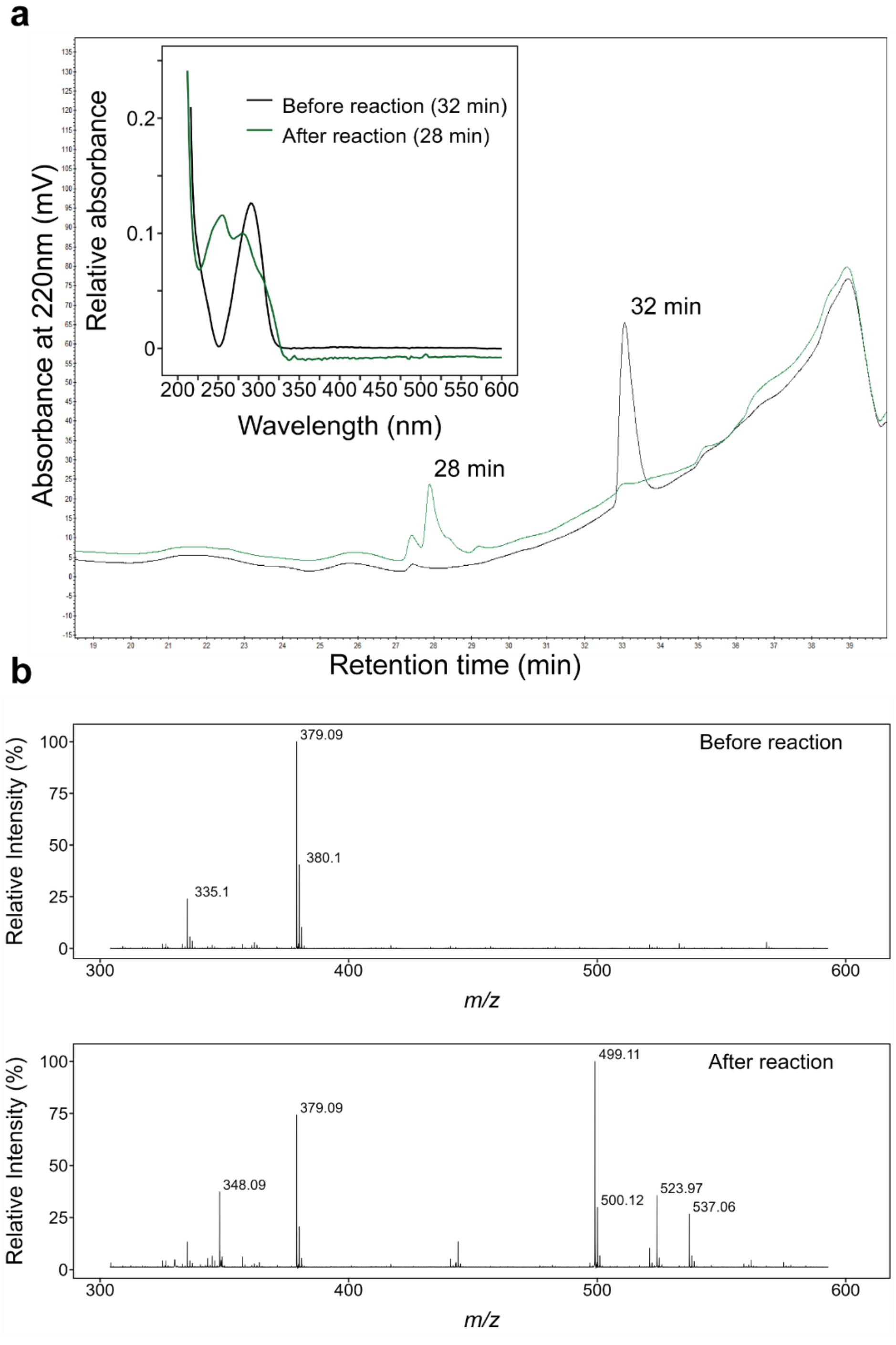

Figure S5. Preparation and characterization of non-natural guanylylpyridinol **3'**. Compound **3'** was guanylylated from the chemically synthesized pyridinol **2'** by the HcgB catalyzed reaction. (a) HPLC chromatogram of the sample before (black) and after (green) the HcgB reaction. The spectra of 32 min peak (before reaction) and 28 min peak (after reaction) are identical to those of pyridinol **2** and guanylylpyridinol **3**, respectively,[8] which indicated the conversion of pyridinol (**2'**) to guanylylpyridinol (**3'**) by the HcgB reaction (inset). The reaction product in the 28 min peak was used for *in vitro* biosynthesis. (b) MALDI-TOF-MS spectra of the HcgB catalyzed solution. Only after the reaction, the mass peak at 499.11 *m/z* was observed, which corresponds to **3'** (calculated mass, $C_{18}H_{23}N_6O_9P_1$, $[M+H]^+$ = 499.1337 *m/z*). The purified compound **3'** shows a 497.1177 *m/z* in HRES-LC-MS (calculated mass, $C_{18}H_{23}N_6O_9P_1$, $[M–H]^–$ = 497.1191 *m/z*). The 379.09 *m/z* peak observed before and after HcgB reaction was also detected in the sample containing only the matrix compounds.

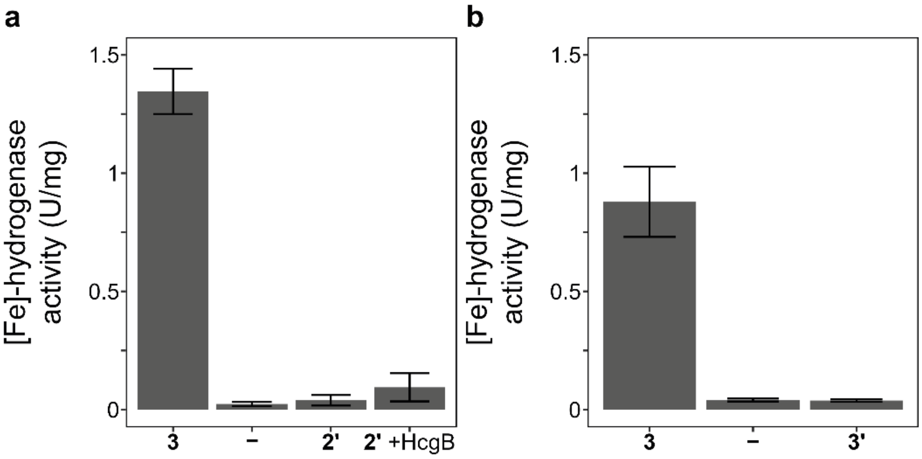

Figure S6. *In vitro* biosynthesis from the decarboxylated pyridinol precursors **2'** and **3'** (for the structure, see Scheme 1 in the main text). (a) *In vitro* biosynthesis activity obtained from **2'** in the absence or presence of HcgB. (b) *In vitro* biosynthesis activity obtained from **3'**. In the abscissa, the precursors and the enzyme added in the assay are shown. As a positive control, *in vitro* biosynthesis activity obtained from **3** is shown. As a negative control, an assay without precursor was tested (–).

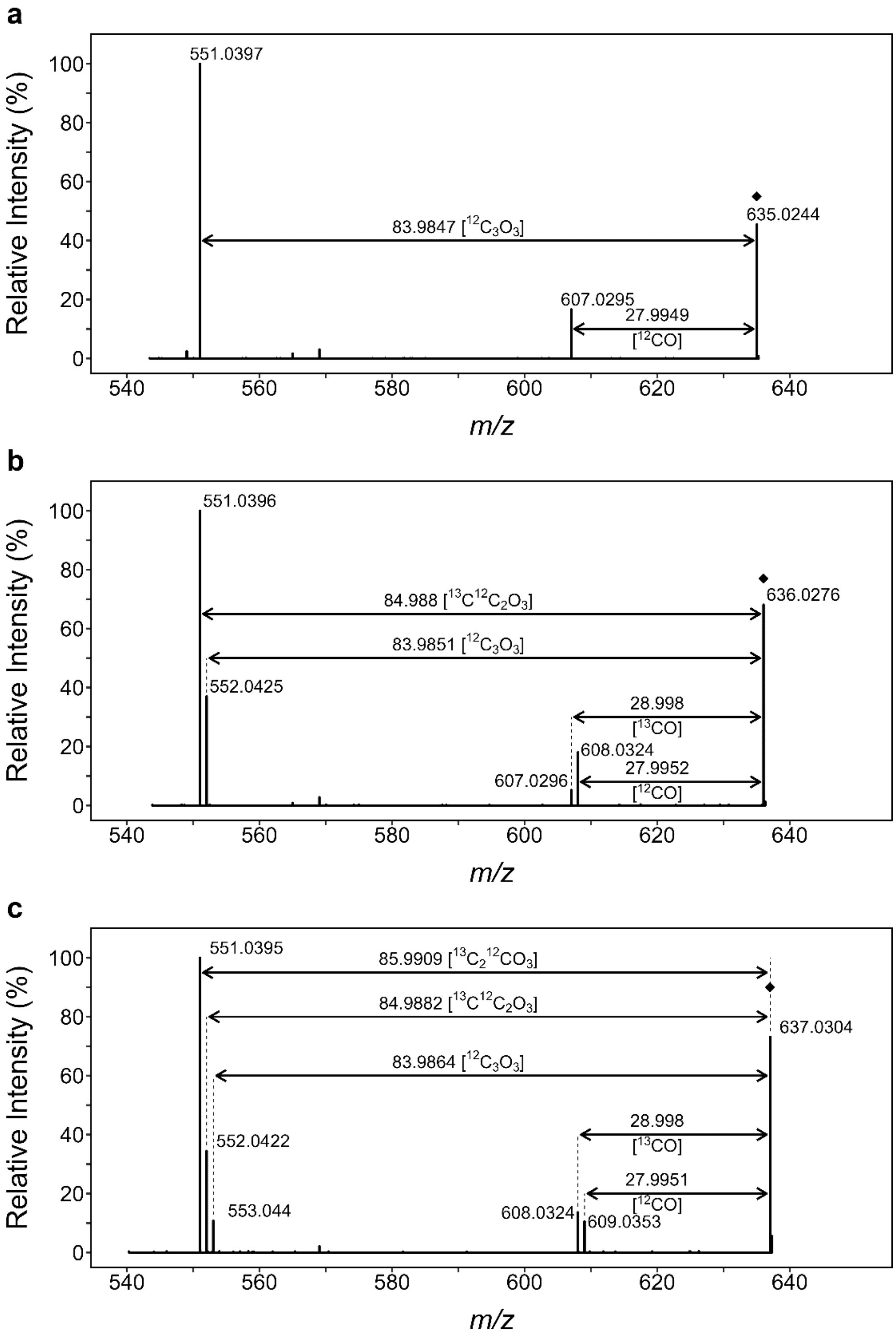

Figure S7. MS/MS spectra of the FeGP cofactor MS species with different ^{13}C content. (a) MS/MS spectrum of the FeGP cofactor containing no ^{13}C ($C_{21}H_{21}N_6O_{12}P_1Fe_1$, [M-H]$^-$ calculated = 635.0232 *m/z*). (b) MS/MS spectrum of the FeGP cofactor containing one ^{13}C ($^{13}C^{12}C_{20}H_{21}N_6O_{12}P_1Fe_1$, [M-H]$^-$ calculated = 636.0265 *m/z*). (c) MS/MS spectrum of the FeGP cofactor containing two ^{13}C ($^{13}C_2{}^{12}C_{19}H_{21}N_6O_{12}P_1Fe_1$, [M-H]$^-$ calculated = 637.0299 *m/z*). For all spectra, ions in the range of [M-H]$^-$ ± 5 ppm were selected for collision-induced dissociation. Diamonds indicates the corresponding precursor ions. Calculated masses for the lost fragments are: ^{12}CO (27.9949), ^{13}CO (28.9982), $^{12}C_3O_3$ (83.9847), $^{13}C^{12}C_2O_3$ (84.9881) and $^{13}C_2{}^{12}CO_3$ (85.9915). These MS/MS data indicated that [^{13}C]-CO and/or [^{13}C]-acyl ligands lost by fragmentation as CO molecules.

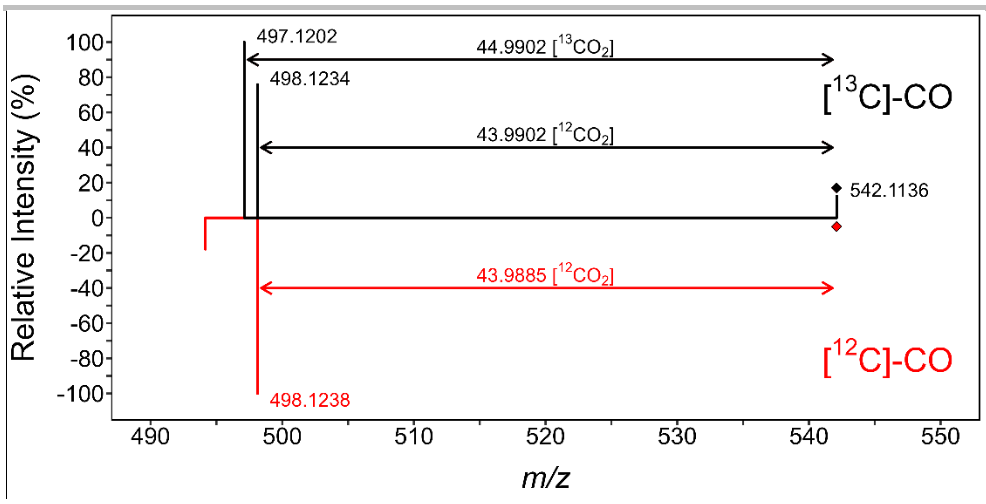

Figure S8. MS/MS spectra of **3** obtained by light decomposition of the FeGP cofactor produced in *in vitro* biosynthesis. (Black) MS/MS spectrum of M1 of **3** ($^{13}C_1C_{18}H_{22}N_6O_{11}P_1$, $[M\text{-}H]^-$ calculated = 542.112324) prepared by decomposition of [^{13}C]-CO labelled FeGP cofactor from *in vitro* biosynthesis. The 497.1202 and 498.1234 *m/z* fragments are produced by decarboxylation of **3** by removal of [^{13}C]-CO_2 and [^{12}C]-CO_2, respectively. The presence of a 497.1202 *m/z* fragment indicates that the ^{13}C label is present at the carboxy group. (Red) MS/MS spectrum of M1 of **3** from non-labelled FeGP cofactor under the gas phase containing [^{12}C]-CO. Decarboxylation of M1 yields only 498.1238 *m/z* fragment by removal of [^{12}C]-CO_2, which indicates no ^{13}C labeling of the carboxy group. Ions in the range of [M-H] ± 5 ppm were selected for collision-induced dissociation. Diamonds designate the corresponding precursor ion. Calculated mass for [^{12}C]-CO_2 is 43.9898 and for [^{13}C]-CO_2 is 44.9932. As the carboxy group of **3** is the hydrolyzed product of the acyl ligand, these MS/MS data indicates that the acyl ligand is partially enriched with [^{13}C]-CO, which could be the result of scrambling.

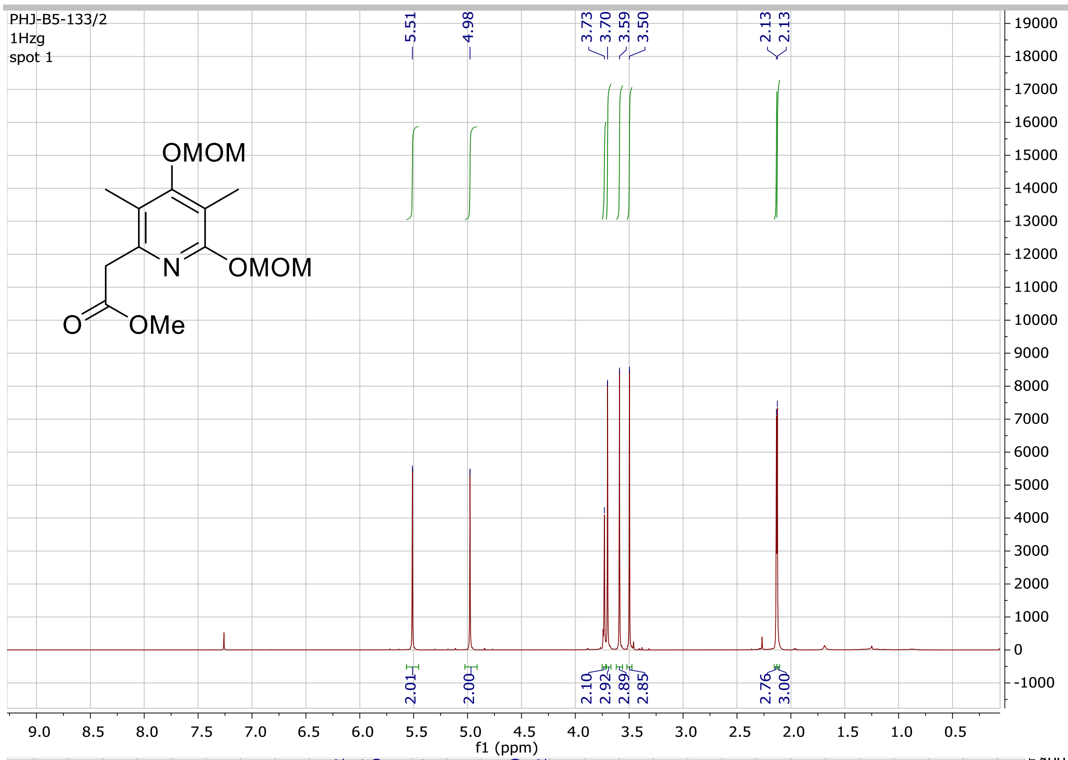

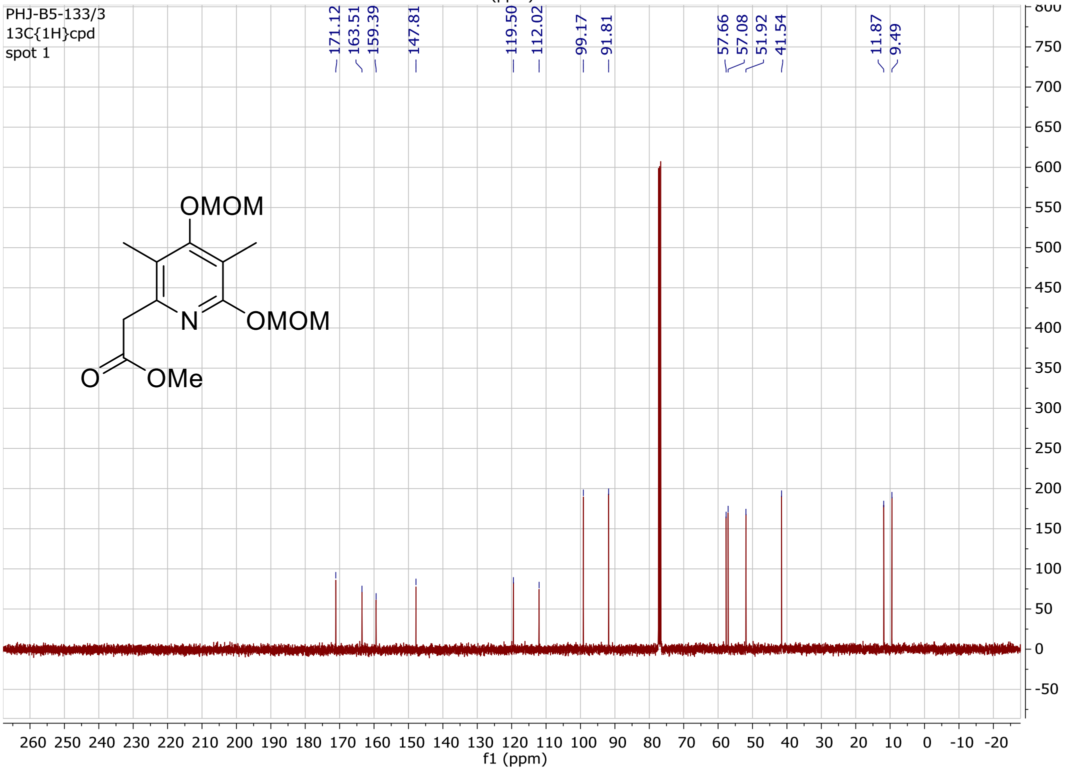

Figure S9. $^{1}H/^{13}C$-NMR trace of **P3**.

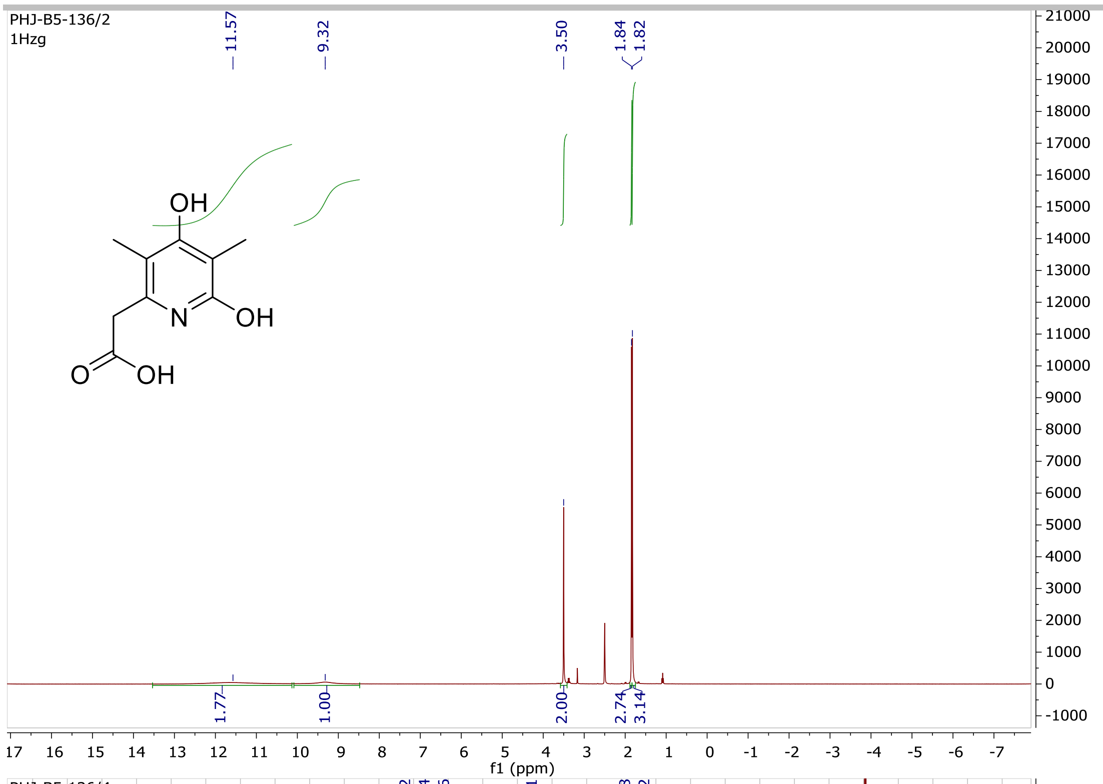

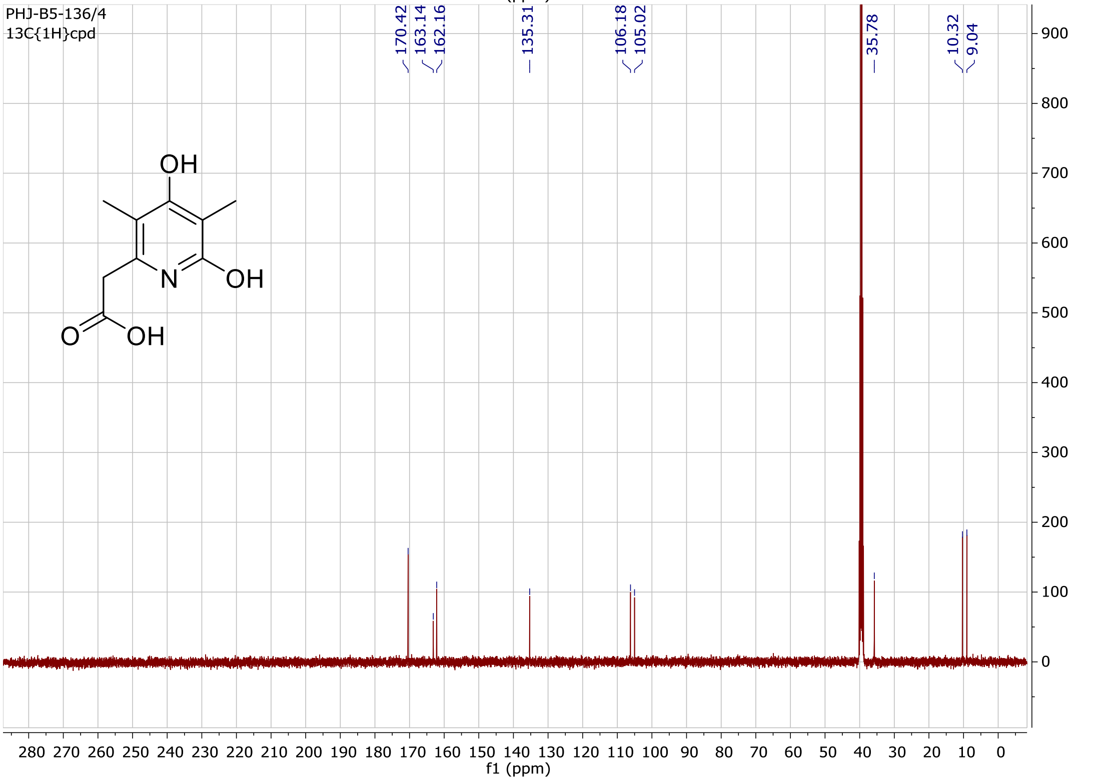

Figure S10. $^{1}H/^{13}C$-NMR trace of **2**.

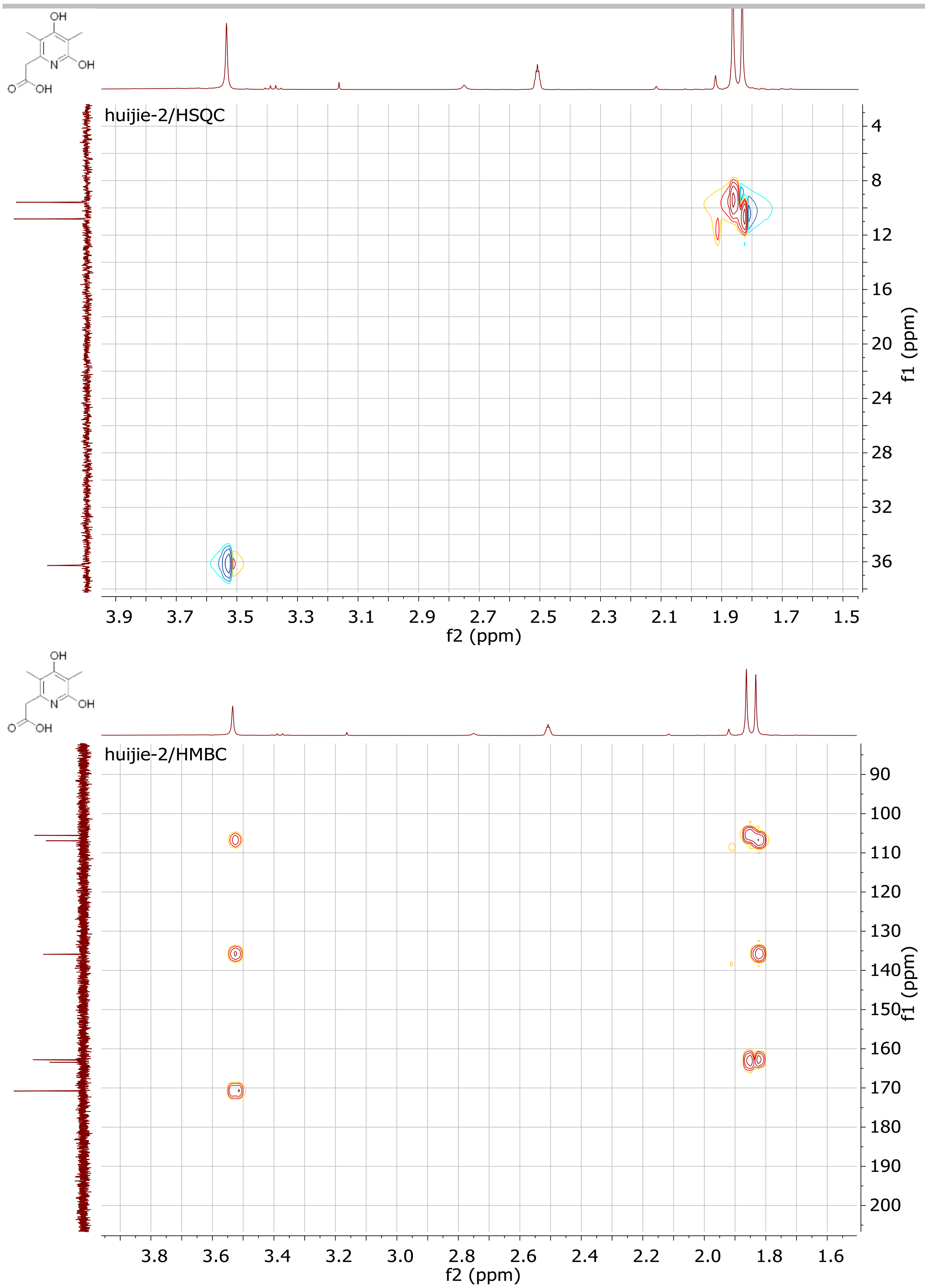

Figure S11. HSQC (top) and HMBC (bottom) NMR spectra of **2**.

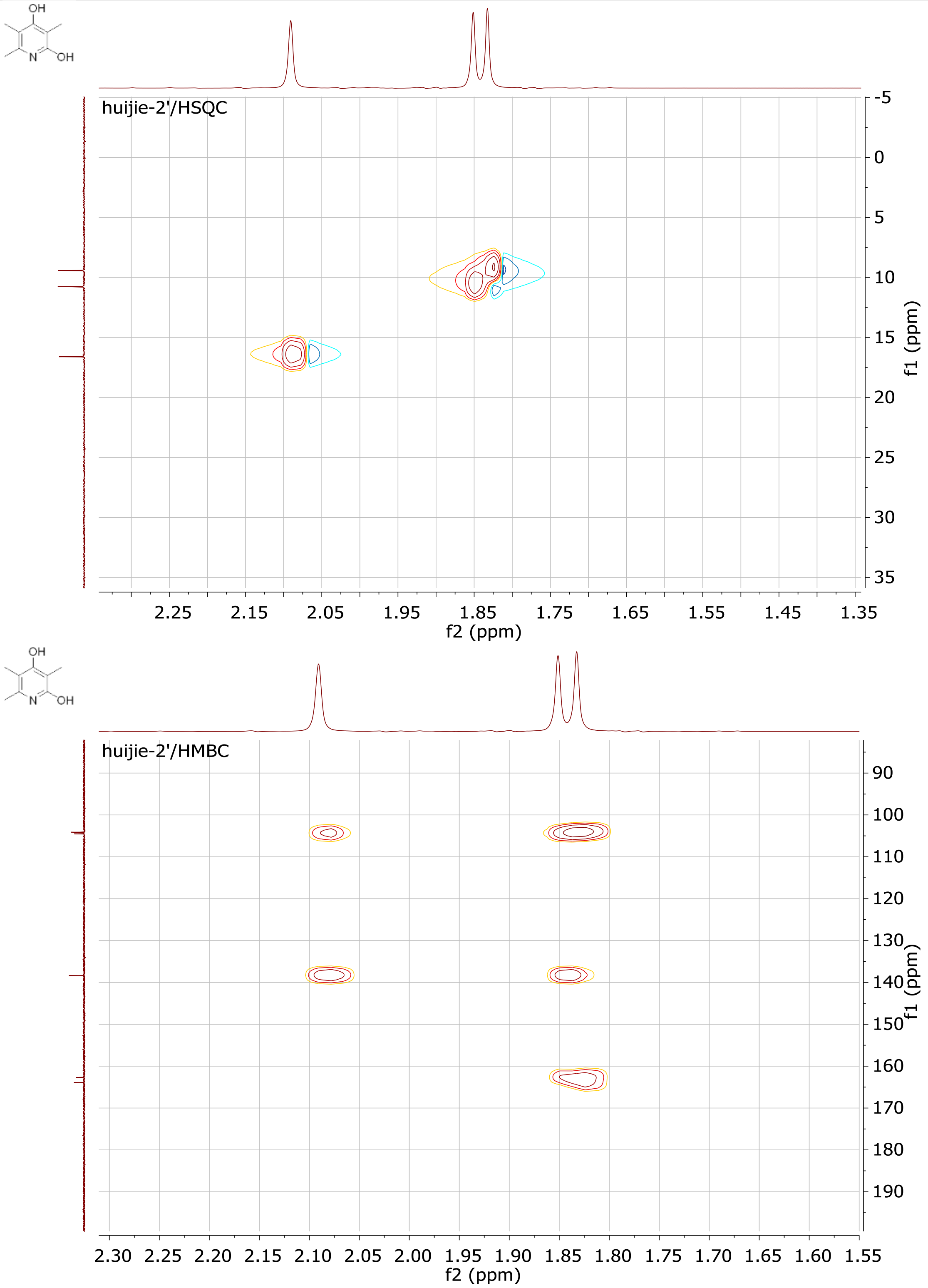

Figure S12. HSQC (top) and HMBC (bottom) NMR spectra of **2'**.

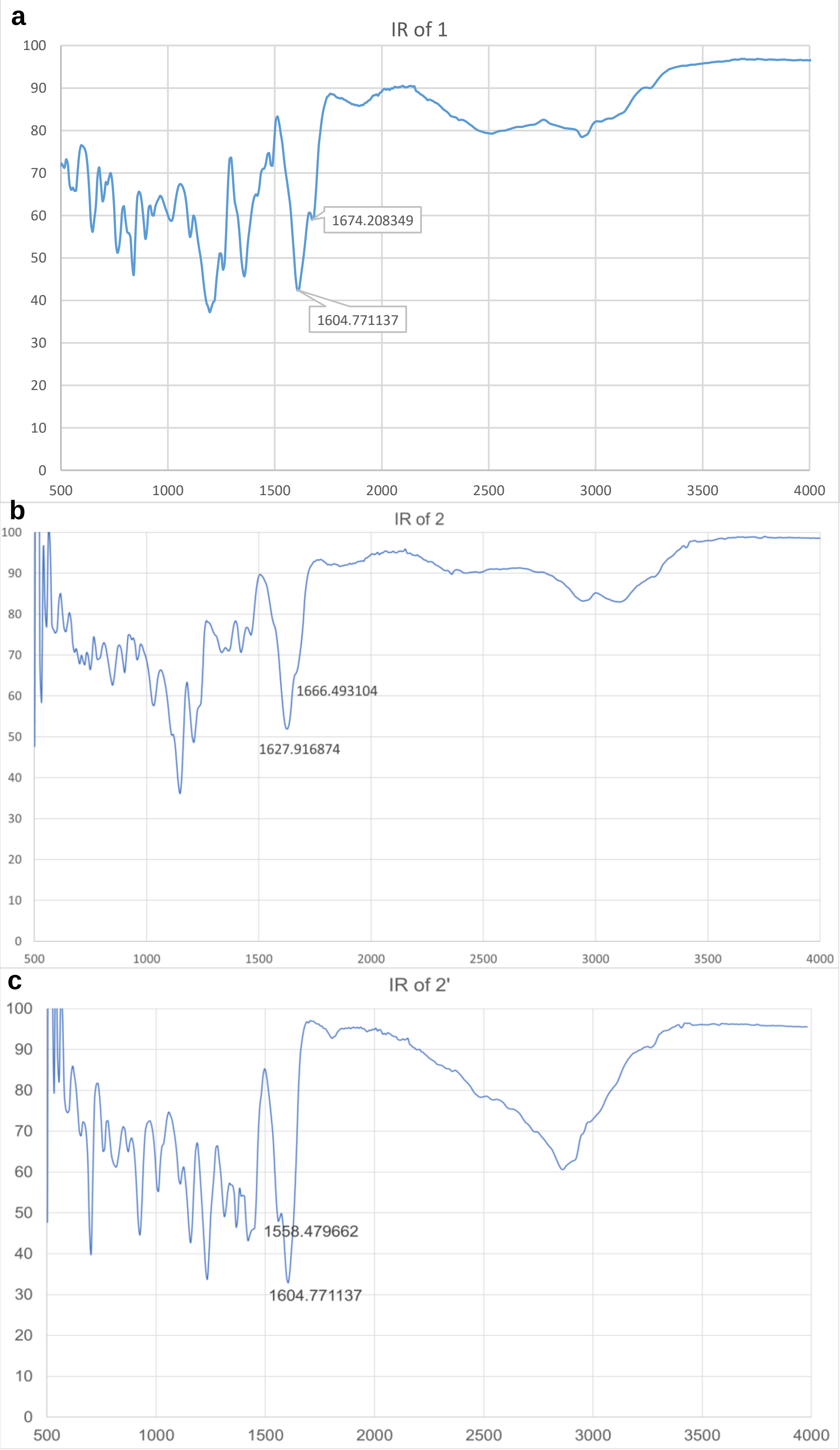

Figure S13. IR spectra of **1** (**a**), **2** (**b**) and **2’** (**c**).

Table S1. Detection of Hcg proteins by proteomics in the *M. maripaludis* Δ*hcgB*Δ*hcgC* strain.

Hcg protein (Accession number)	Coverage	Number of peptides (unique peptides)
**HcgA** (WP_011170070.1)	91.79%	41 (10)
**HcgB** (WP_011171441.1)	-	-
**HcgC** (WP_011171442.1)	-	-
**HcgD** (WP_011169997.1)	86.99%	25 (13)
**HcgE** (WP_013999640.1)	81.86%	27 (6)
**HcgF** (WP_011170879.1)	-	-
**HcgG** (WP_011170069.1)	78.04%	48 (18)
**Hmd** (WP_011170071.1)	84.18%	43 (28)

PCR experiments confirmed that the genome of the Δ*hcgB*Δ*hcgC* strain contains the *hcgF* gene. HcgF was not detected by this proteome analysis. It was previously reported that deletion of *hcgF* resulted in a delay of growth, which was observed in the mutants lacking functional [Fe]-hydrogenase, while the phenotype was complemented by expression vector containing *hcgF*.[9] These experiments indicated that HcgF is functional in *M. maripaludis*.

References

[1] T. Fujishiro, L. Bai, T. Xu, X. Xie, M. Schick, J. Kahnt, M. Rother, X. Hu, U. Ermler, S. Shima, *Angew. Chem. Int. Ed.* **2016**, *55*, 9648-9651.
[2] O. Pilak, B. Mamat, S. Vogt, C. H. Hagemeier, R. K. Thauer, S. Shima, C. Vonrhein, E. Warkentin, U. Ermler, *J. Mol. Biol.* **2006**, *358*, 798-809.
[3] W. T. McElroy, P. DeShong, *Org. Lett.* **2003**, *5*, 4779-4782.
[4] S. Shima, M. Schick, H. Tamura, *Methods Enzymol.* **2011**, *494*, 119-137.
[5] W. E. Balch, G. E. Fox, L. J. Magrum, C. R. Woese, R. S. Wolfe, *Microbiol. Rev.* **1979**, *43*, 260-296.
[6] F. Long, L. Wang, B. Lupa, W. B. Whitman, *Archaea* **2017**, *2017*, 7046026.
[7] P. Millard, B. Delépine, M. Guionnet, M. Heuillet, F. Bellvert, F. Létisse, *Bioinformatics* **2019**, *35*, 4484-4487.
[8] S. Shima, E. J. Lyon, M. S. Sordel-Klippert, M. Kauß, J. Kahnt, R. K. Thauer, K. Steinbach, X. L. Xie, L. Verdier, C. Griesinger, *Angew. Chem. Int. Ed.* **2004**, *43*, 2547-2551.
[9] T. J. Lie, K. C. Costa, D. Pak, V. Sakesan, J. A. Leigh, *FEMS Microbiol. Lett.* **2013**, *343*, 156-160.

Author Contributions

S.Sh. directed the research. S.Sc, F.J.A.-G and S.Sh. designed the experiments. S.Sc and F.J.A.-G performed sample preparation, *in vitro* biosynthesis and biochemical analysis. H.-J.P. and X.H. performed chemical synthesis. J.K performed proteome analysis and MALDI-TOF-MS of **3'**. G.A. and N.P. performed HRES-LC-MS analysis. C.K. performed genetics of *M. maripaludis*. S.Sc. J.A.-G and S.Sh. analyzed the data. S.Sc. and S.Sh. wrote the manuscript with contributions from other authors.
